# A novel susceptibility locus in *MST1* and gene‐gene interaction network for Crohn's disease in the Chinese population

**DOI:** 10.1111/jcmm.13530

**Published:** 2018-02-14

**Authors:** William K.K. Wu, Rui Sun, Tao Zuo, Yuanyuan Tian, Zhirong Zeng, Jeffery Ho, Justin C.Y. Wu, Francis K.L. Chan, Matthew T.V. Chan, Jun Yu, Joseph J.Y. Sung, Sunny H. Wong, Maggie H. Wang, Siew C. Ng

**Affiliations:** ^1^ State Key Laboratory of Digestive Diseases Institute of Digestive Diseases and Department of Medicine & Therapeutics LKS Institute of Health Sciences CUHK Shenzhen Research Institute The Chinese University of Hong Kong Hong Kong; ^2^ Department of Anaesthesia and Intensive Care The Chinese University of Hong Kong Hong Kong; ^3^ The Jockey Club School of Public Health and Primary Care The Chinese University of Hong Kong Hong Kong; ^4^ Department of Gastroenterology The First Affiliated Hospital of Sun Yat‐Sen University Guangzhou China

**Keywords:** Crohn's disease, fine mapping, gene‐gene interactions, MST1, next‐generation sequencing

## Abstract

The incidence of Crohn's disease is increasing in many Asian countries, but considerable differences in genetic susceptibility have been reported between Western and Asian populations. This study aimed to fine‐map 23 previously reported Crohn's disease genes and identify their interactions in the Chinese population by Illumina‐based targeted capture sequencing. Our results showed that the genetic polymorphism A>G at rs144982232 in *MST1* showed the most significant association (*P *=* *1.78 × 10^−5^; odds ratio = 4.87). *JAK2* rs1159782 (T>C) was also strongly associated with Crohn's disease (*P *=* *2.34 × 10^−4^; odds ratio = 3.72). Gene‐gene interaction analysis revealed significant interactions between *MST1* and other susceptibility genes, including *NOD2*,* MUC19* and *ATG16L1* in contributing to Crohn's disease risk. Main genetic associations and gene‐gene interactions were verified using ImmunoChip data set. In conclusion, a novel susceptibility locus in *MST1* was identified. Our analysis suggests that *MST1* might interact with key susceptibility genes involved in autophagy and bacterial recognition. These findings provide insight into the genetic architecture of Crohn's disease in Chinese and may partially explain the disparity of genetic signals in Crohn's disease susceptibility across different ethnic populations by highlighting the contribution of gene‐gene interactions.

## 1 INTRODUCTION

Crohn's disease is one of the two major forms of inflammatory bowel diseases (IBD) characterized by chronic and relapsing inflammation in the gastrointestinal tract. Crohn's disease has long been thought to be uncommon in Asian countries. However, the incidence of Crohn's disease has been rapidly increasing in Asian countries as seen in many recent epidemiological studies.^1^, ^2^ In this regard, China has the highest incidence of IBD in Asia within the Asia‐Pacific Crohn's and Colitis Epidemiologic Study Group.^1^

Genetic susceptibility, gut microbiota and environmental factors act synergistically in the pathogenesis of Crohn's disease. Although more than 140 susceptibility loci of Crohn's disease in Caucasians have been identified by genome‐wide association studies (GWASs) and meta‐analyses,^4^, ^5^, ^6^, ^7^, ^8^, ^9^ considerable differences in genetic susceptibility to Crohn's disease have been reported between Western and Asian populations. Moreover, the heritability of Crohn's disease in Asian populations has not been fully explained.^5^, ^6^ In particular, the well‐established Caucasian Crohn's disease susceptibility genes, such as *NOD2*,* ATG16L1* and *PTPN*, showed a lack of association in the Asian populations.^6^, ^8^, ^10^, ^11^, ^12^, ^13^, ^14^, ^15^ Inconsistent results on *IL23R* and *IRGM* were also reported.^16^, ^17^ Recent genetic studies in Korean and Japanese populations further revealed new Crohn's disease susceptibility loci (eg rs11235604 in *ATG16L2* and rs7329174 in *ELF1*) that were not significantly associated with disease status in Western populations.^18^, ^19^, ^20^ This may be in part related to heterogeneity in effect size (eg *TNF‐SF15* and *ATG16L*), differences in risk allele frequency in some of the loci (eg *CARD15/NOD2*) or altered gene‐microbiota and gene‐gene interactions across different populations.^21^ Collectively, these findings underpinned different genetic architectures in different ethnicities in determining genetic risk for Crohn's disease.

The impact of new loci underlying susceptibility to Crohn's disease cannot be determined until causal variants are identified by fine mapping via directed sequencing. Moreover, it is imperative to determine whether Crohn's disease susceptibility genes identified in Europeans are also associated with disease state in non‐European ancestry populations.^22^ To address whether genes previously reported in Caucasian populations contribute to Crohn's disease in the Chinese population and their effect sizes, we performed fine‐mapping analysis using next‐generation targeted capture sequencing. Moreover, as interactions among multiple genes could impact on the patients’ disease phenotype, we aimed to identify interactions among the targeted captured genes to provide insight into the genetic of Crohn's disease.

## 2 METHODS

### 2.1 Study participants

Crohn's disease patients of Han Chinese ethnicity and healthy individuals were recruited at the Prince of Wales Hospital, Hong Kong, and the First Affiliated Hospital of Sun Yat‐Sen University, Guangzhou. Both hospitals are geographically located in the Guangdong Province of China. Inclusion criteria of cases included (*i*) age >18 years old and (*ii*) diagnosis of Crohn's disease established in accordance with clinical, radiological, endoscopic and histological features criteria. Inclusion criteria of controls included (*i*) age >18 years and (*ii*) asymptomatic individuals participating in colonoscopy screening or healthy volunteers or students from the Chinese University of Hong Kong. The study was prospectively reviewed and approved by the Joint CUHK‐NTEC Clinical Research Ethics Committee and the clinical ethics committee of Sun Yat‐Sen University. All participants had provided written informed consents. Controls were excluded if they had previously been diagnosed with IBD or if they had one or more first‐ or second‐degree relative with IBD. Clinical phenotype data were collected and stored in a database: age, sex, family history, smoking history, surgery and date of first surgery, extraintestinal diseases, disease location and behaviour (Montreal classification).

### 2.2 Targeted gene capture and next‐generation sequencing

The chip‐based gene capture technology coupled with next‐generation sequencing was employed for comprehensive genotyping of 23 Crohn's disease susceptibility genes and their promoters. Genomic DNA was extracted from blood lymphocytes (250 μL) of Crohn's disease patients and healthy individuals using Gentra Puregene Blood Kit (Gentra Systems, Inc., Minneapolis, MN) and stored at −20°C in the Prince of Wales Hospital. Twenty micrograms of genomic DNA from each sample was sheared by nebulizer (Roche Applied Science, Hong Kong) to fragments around 500 bp. After ligation with linkers at both ends, small fragments <300 bp were removed with AMPure DNA purification beads (Agencourt, Beverly, MA). The linker‐ligated DNA fragments were then hybridized to the custom‐designed NimbleGen Sequence Capture 2.1M Array and the enriched captured DNA fragments were eluted from the array and amplified by ligation‐mediated PCR. Quantitative PCR was used to estimate the magnitude of enrichment. Twenty‐three genes captured for next‐generation sequencing are listed as follows: *PTPN22*,* IL23R*,* ITLN1*,* ATG16L1*,* PTGER4*,* MST1*,* IRGM*,* IL12B*,* CDKAL1*,* CCR6*,* JAK2*,* TNFSF15*,* ZNF365*,* NKX2‐3*,* C11orf30*,* LRRK2*,* MUC19*,* NOD2*,* ORMDL3*,* STAT3*,* PTPN2*,* ICOSLG* and *VDR*.

The captured DNA fragments were first randomly ligated by DNA ligase to sizes ranged from 1 to 8 kb, then sheared to 200 bp on average, and finally ligated with Illumina‐compatible adapters and subject to standard library preparation. Resulting DNA libraries were sequenced on Illumina 2000 with the target sequencing depth of 50×, which was more than sufficient for genotyping heterozygous loci with high confidence. Real‐time image analysis and base calling were performed by the Genome Analyzer Pipeline version 1.3.1 using standard parameters. Reads with ≥ 12‐bp adapter or linker sequences or reads <29 bp, or with >6 missing bases, or 40 continuous identical bases were discarded. SOAP aligner was used to align the remaining reads to the human reference genome (human NCBI Build 36) with maximum two mismatches. Only unique matched reads were retained. A Bayesian statistics‐based algorithm was used for base calling.

### 2.3 ImmunoChip data set

The design and genotyping of the ImmunoChip have been previously described.^21^ In brief, the ImmunoChip is an Illumina Infinium microarray comprising 196 524 single nucleotide polymorphisms (SNPs) and small indel markers selected based on results from GWASs of 12 different immune‐mediated diseases. The ImmunoChip enables replication of all nominally associated SNPs (*P* < .001) from the index GWAS scans and fine mapping of 186 loci associated at genome‐wide significance with at least 1 of the 12 index immune‐mediated diseases. The chip also contains around 3000 SNPs added as part of the Wellcome Trust Case Control Consortium 2 (WTCCC2) project replication phase. The genotype data were extracted for 531 Hong Kong Chinese subjects on the ImmunoChip data set. Quality control was performed as described.^21^ The cohort includes 235 controls and 531 IBD cases, including 388 patients with Crohn's disease.

### 2.4 Statistical analysis

The SNPs from targeted sequencing had low to rare minor allele frequencies (MAFs). The sequence kernel association test (SKAT) is an effective method to detect association of the sequencing data to disease phenotypes.^23^, ^24^ The method uses a linear mixed model and performs variance component score test.^24^ For epistasis evaluation, a robust *W*‐test was used to evaluate SNP‐SNP interactions.^25^ The *W*‐test is testing for the difference in genotype distributions formed by a SNP pair in case and control groups. The test follows a chi‐squared distribution of which the degrees of freedom is bootstrap‐estimated from the data. Therefore, the method is able to correct for bias in distributions due to complicated genetic architecture and return robust estimates.^25^ The SKAT and *W*‐test were conducted using R packages.^23^, ^25^ The LocusZoom tool was used to draw SNPs Manhattan plot in a specific region and provided a detailed view of the *P*‐value distribution within a gene.^26^ A SNP or an interaction pair was significant if its *P*‐value was smaller than Bonferroni‐corrected alpha of 5%. Expression quantitative trait loci (eQTL) analysis was carried out using the Genotype‐Tissue Expression database.^27^

### 2.5 Power calculation

The power of an association study depends on the sample size, effect size of a variant and its allele frequency. Assuming findings from a previously validated SNP (rs2241880) in *ATG16L1* with an odds ratio of 0.69 and a minor allele frequency of 45%, our study had 86.2% power to detect such a variant with an α‐error rate of 5%. Alternatively, our study had at least 80% power to detect a variant with odds ratio of 1.5 at a MAF of 20%.

## 3 RESULTS

### 3.1 Patient characteristics, quality control and SNP calling

A total of 262 patients with Crohn's disease and 323 controls were included. The mean age was 43.6 and 55.9 years in the case and control groups, respectively. About half of the subjects were female (45.9%). Table S1 summarizes the basic characteristics of the cases and controls. DNA samples were collected from all patients for targeted capture sequencing, generating a genotype data set of 2046 SNPs. Four subjects were excluded because of empty data files. Targeted capture of all DNA samples was completed with an average sequencing depth (on target) of >50 and a coverage of >99.7% (Table S2). When calling the genotype, missing value was coded if genotype quality was less than 20. Quality control of the genotype data was conducted, and we excluded samples whereby (*i*) the percentage of missing genotypes was greater than 5%, (*ii*) SNPs had no variance, and (*iii*) *P*‐values of test on Hardy‐Weinberg equilibrium (HWE) were smaller than 0.05 after Bonferroni correction^28^ (Table S3).

### 3.2 Novel associations of *MST1 rs144982232* with Crohn's disease

**Figure 1:**
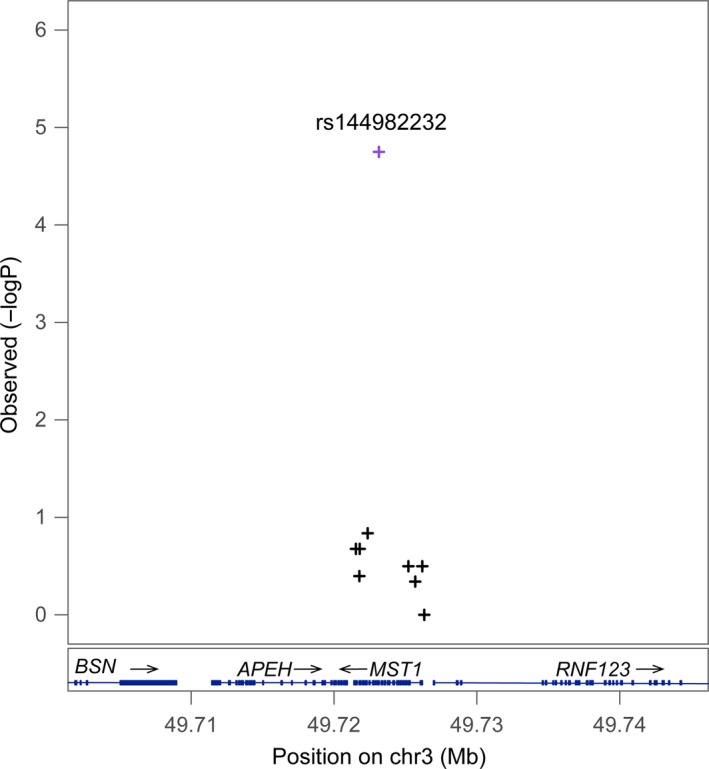
Regional association plot of *MST1*. The A>G polymorphism at rs144982232 corresponding to synonymous H425H increased the risk for Crohn's disease in the Chinese population (*P *=* *1.78E‐05; odd ratios: 4.87). Grey colour indicates that the information of linkage disequilibrium (*r* ^2^ values) for the points was not available in reference genome

**Figure 2:**
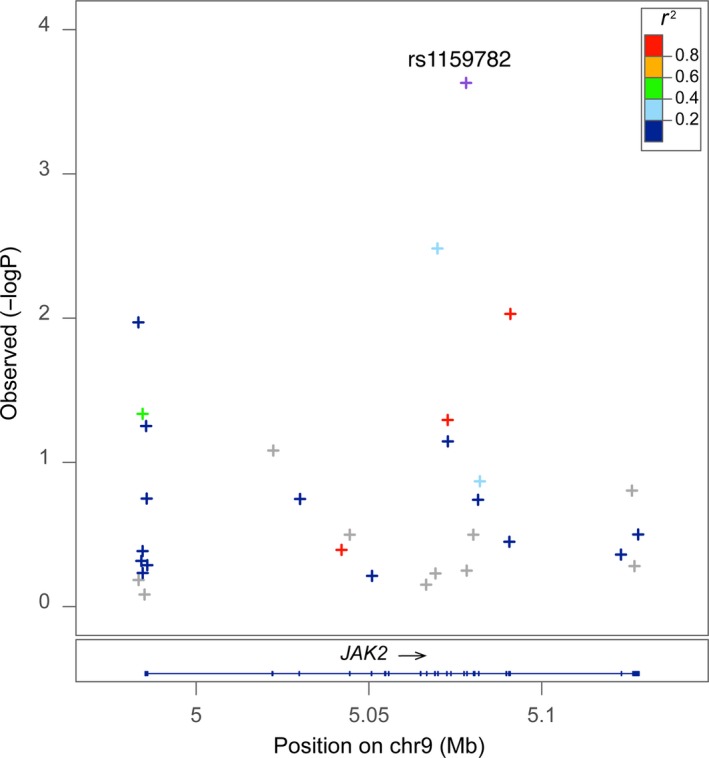
Regional association plot of *JAK2*. Subjects with allele T>C at rs1159782 had a higher risk for Crohn's disease (*P *=* *2.34E‐04, odds ratio: 3.72). The *r* ^2^ was estimated by the LocusZoom software from HapMap Phase II JPT + CHB population. It measures the linkage disequilibrium of each SNP with the most significant SNP

Sequence kernel association test analysis identified one locus, namely the rs144982232 in *MST1* (A>G, *P *=* *1.78 × 10^−5^, odds ratio = 4.87), which was significantly associated with Crohn's disease after controlling for multiple testing by Bonferroni method (Tables 1 and S6). The susceptibility to the disease was 4.87 higher for individuals with a G allele at this locus than those with an A allele (odds ratio = 4.87). The regional association plot of *MST1* is shown in Figure 1. There were 26 SNPs sequenced in this gene in our fine‐mapping study. The distribution of three genotypes (GG, AA and GA) at *MST1* rs144982232 was in accordance with HWE. Two more SNPs showing strong effect were rs1159782 in *JAK2* (T>C, *P *=* *2.34 × 10^−4^, odds ratio = 3.72; Figure 2) and rs2111234 in *NOD2* (G>A, *P *=* *7.59 × 10^−4^, odds ratio = 5.09). The large odds ratios indicated a strong risk effect in the *MST1*,* JAK2* and *NOD2* gene to Crohn's disease.

**Table 1:** Top 10 SNPs identified by SKAT among 23 Crohn's disease susceptibility genes. The total number of SNPs is 2046, and the Bonferroni‐corrected significance threshold is 2.4 × 10^−5^

Rank	Chr	Pos	SNP	*P*‐value	Gene	Description	MAF	Odds ratio	95% CI
1	Chr3	49723141	rs144982232	1.78E‐05	*MST1*	A>G	0.038	4.87	2.25, 10.54
2	Chr9	5078117	rs1159782	2.34E‐04	*JAK2*	T>C	0.053	3.72	2.03, 6.82
3	Chr16	50734033	rs2111234	7.59E‐04	*NOD2*	G>A	0.034	5.09	2.24, 11.58
4	Chr9	117569046	rs7848647	1.46E‐03	*TNFSF15*	T>C	0.448	0.69	0.55, 0.87
5	Chr10	64418656	rs7915131	1.34E‐03	*ZNF365*	C>T	0.343	0.68	0.54, 0.87
6	Chr1	114377148	rs1970559	3.29E‐03	*PTPN22*	T>C	0.041	0.39	0.21, 0.72
7	Chr1	114396955	rs2476602	4.09E‐03	*PTPN22*	G>A	0.039	0.38	0.20, 0.73
8	Chr10	64426056	rs4746516	2.03E‐03	*ZNF365*	G>T	0.200	0.63	0.47, 0.84
9	Chr9	5069837	rs7869668	3.29E‐03	*JAK2*	G>A	0.417	1.44	1.15, 1.82
10	Chr10	64418089	rs10822044	4.33E‐03	*ZNF365*	T>C	0.231	0.68	0.52, 0.89

MAF, minor allele frequencies; SKAT, sequence kernel association test; SNP, single nucleotide polymorphisms.

### 3.3 Interactions of *MST1* with *MUC19*,* JAK2*,* VDR* and other Crohn's disease susceptibility genes

**Figure 3:**
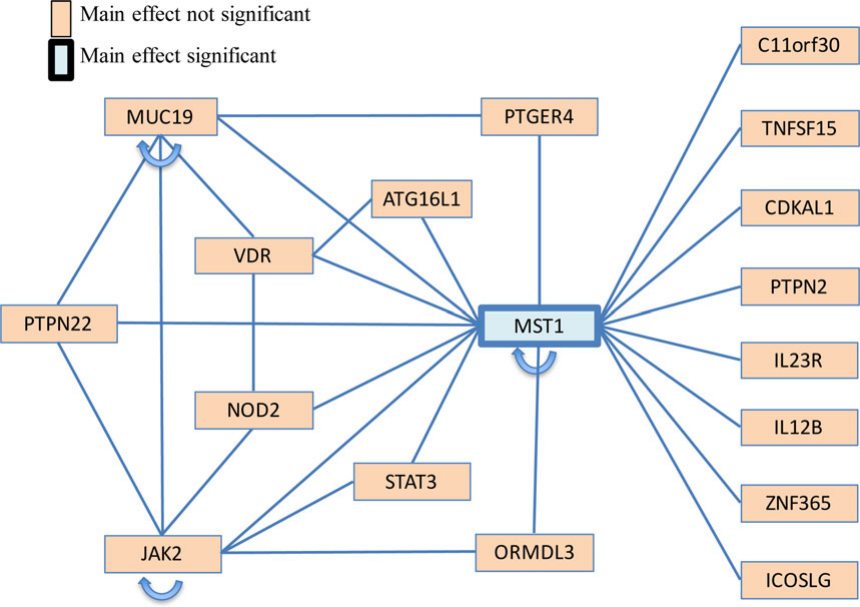
Gene‐gene interaction network visualizing the results of *W*‐test. *MST1* had extensive interactions with other Crohn's disease susceptibility genes. *JAK2*,*NOD2*,*MUC19* and *VDR* also interacted widely. An arrow indicates interactions between two or more SNPs within the same gene

A robust and powerful epistasis analysis tool known as *W*‐test was performed to detect SNP‐SNP interactions.^25^ From the total 2046 SNPs in this study, 202 SNPs had *P*‐value less than 0.1 and passed the first‐stage filtering. Among these SNPs, 20 301 SNP pairs were formed, and a total number of 95 pairs had *P*‐values passed the Bonferroni‐corrected significance level at 2.46 × 10^−6^. Top interacting pairs identified are shown in Tables 2 and S7. The significant SNP pairs producing an 18‐gene interaction network are visualized in Figure 3, in which *MST1*,* MUC19*,* JAK2* and *VDR* play central roles. Interestingly, except *MST1*, none of the 18 genes showed significant main effect. The top pairs include interactions of *MST1*‐*JAK2* (A>G at rs144982232, T>C at rs1159782, *P *=* *9.44 × 10^−11^, odds ratio = 4.34), *MST1‐NOD2* (A>G at rs144982232, G>A at rs2111234, *P *=* *1.79 × 10^−9^, odds ratio = 4.69) and *MST1‐MUC19* (A>G at rs144982232, T>C at rs116937891, *P *=* *1.01 × 10^−8^, odds ratio = 4.89). For the interaction pair *MST1‐JAK2*, the susceptibility to Crohn's disease was 4.34 higher for genotypes with allele G at rs144982232 in *MST1* or with allele C at rs1159782 in *JAK2* than those with allele A at rs144982232 and T at rs1159782 (odds ratio = 4.34).

**Table 2:** Top 20 SNP‐SNP interactions among 95 significant pairs in 23 genes identified by *W*‐test. A *P*‐value < 2.46 × 10^−6^ was considered statistically significant

Rank	SNP1	Chr	Position1	Gene1	SNP1 description	SNP2	Chr	Position2	Gene2	SNP2 description	OR	95% CI	MAF1	MAF2	*P*‐value
1	rs144982232	Chr3	49723141	*MST1*	A>G	rs1159782	Chr9	5078117	*JAK2*	T>C	4.34	2.50, 7.52	0.04	0.05	9.44E‐11
2	rs144982232	Chr3	49723141	*MST1*	A>G	rs2111234	Chr16	50734033	*NOD2*	G>A	4.69	2.55, 8.64	0.04	0.03	1.79E‐09
3	rs144982232	Chr3	49723141	*MST1*	A>G	rs116937891	Chr12	40815261	*MUC19*	T>C	4.89	2.48, 9.62	0.04	0.01	1.01E‐08
4	rs144982232	Chr3	49723141	*MST1*	A>G	rs11564247	Chr12	40821478	*MUC19*	T>C	5.71	2.74, 11.91	0.04	0.01	1.34E‐08
5	rs144982232	Chr3	49723141	*MST1*	A>G	rs80205770	Chr12	40823230	*MUC19*	A>G	3.50	2.05, 5.98	0.04	0.03	2.15E‐08
6	rs144982232	Chr3	49723141	*MST1*	A>G	rs56191322	Chr1	114362437	*PTPN22*	A>G	5.12	2.53, 10.37	0.04	0.01	3.06E‐08
7	rs144982232	Chr3	49723141	*MST1*	A>G	rs191850264	Chr12	40920180	*MUC19*	G>A	4.99	2.46, 10.12	0.04	0.01	3.11E‐08
8	rs144982232	Chr3	49723141	*MST1*	A>G	rs2289473	Chr2	234182025	*ATG16L1*	C>T	4.64	2.35, 9.17	0.04	0.01	4.11E‐08
9	rs2111234	Chr16	50734033	*NOD2*	G>A	rs1159782	Chr9	5078117	*JAK2*	T>C	3.82	2.16, 6.76	0.03	0.05	5.76E‐08
10	rs144982232	Chr3	49723141	*MST1*	A>G	rs12370083	Chr12	40816185	*MUC19*	C>A	4.99	2.46, 10.12	0.04	0.01	6.68E‐08
11	rs6687620	Chr1	67648460	*IL23R*	T>C	rs144982232	Chr3	49723141	*MST1*	A>G	4.99	2.46, 10.12	0.01	0.04	6.73E‐08
12	rs144982232	Chr3	49723141	*MST1*	A>G	rs2229829	Chr12	48238607	*VDR*	G>T	4.64	2.35, 9.17	0.04	0.01	8.16E‐08
13	rs144982232	Chr3	49723141	*MST1*	A>G	rs11564248	Chr12	40820570	*MUC19*	C>T	4.86	2.39, 9.86	0.04	0.01	1.14E‐07
14	rs144982232	Chr3	49723141	*MST1*	A>G	rs2291282	Chr17	40498565	*STAT3*	T>C	3.81	2.12, 6.84	0.04	0.02	1.22E‐07
15	rs144982232	Chr3	49723141	*MST1*	A>G	rs9837520	Chr3	49722356	*MST1*	G>A	3.01	1.81, 5.02	0.04	0.03	1.23E‐07
16	rs144982232	Chr3	49723141	*MST1*	A>G	rs78930461	Chr2	234201767	*ATG16L1*	A>G	5.46	2.50, 11.90	0.04	0.003	1.27E‐07
17	rs144982232	Chr3	49723141	*MST1*	A>G		Chr12	48238682	*VDR*	G>A	5.62	2.58, 12.23	0.04	0.003	1.36E‐07
18	rs144982232	Chr3	49723141	*MST1*	A>G	rs7487333	Chr12	40812148	*MUC19*	C>T	3.58	2.02, 6.34	0.04	0.02	1.53E‐07
19	rs144982232	Chr3	49723141	*MST1*	A>G	rs12601611	Chr17	40497828	*STAT3*	C>T	5.78	2.66, 12.56	0.04	0.01	1.63E‐07
20	rs144982232	Chr3	49723141	*MST1*	A>G		Chr5	40679674	*PTGER4*	G>C	5.12	2.44, 10.75	0.04	0.01	1.96E‐07

MAF, minor allele frequencies; SNP, single nucleotide polymorphisms.

### 3.4 *MST1*,* JAK2*,* MUC19* and *VDR* acted in concert with *NOD2* to alter risk for Crohn's disease

**Figure 4:**
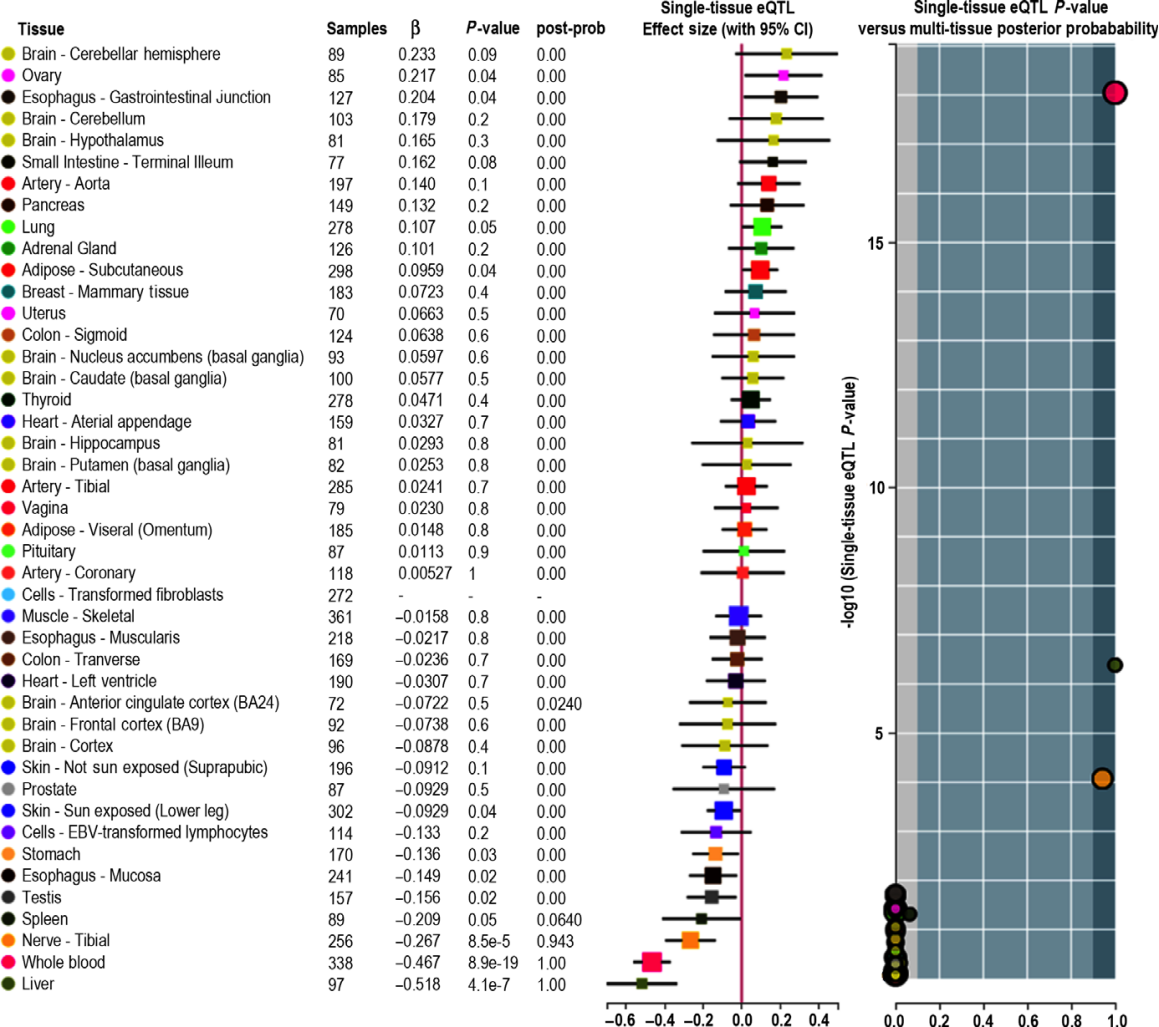
eQTL analysis revealing the association between *NOD2* rs2111234 genotypes and *NOD2* mRNA expression in multiple tissue types

From the regional association plots, rs144982232 in *MST1*, rs1159782 in *JAK2*, rs11564247 in *MUC19* (Figure S1) and rs11574129 in *VDR* (Figure S2) were the most significant SNPs within the genes, but none of these SNPs was associated with gene expression by eQTL analysis (data not shown). Further analysis found that all of these four SNPs interacted with rs2111234 in *NOD2* directly or indirectly. Most interestingly, this polymorphic variant of *NOD2* was strongly associated with altered *NOD2* gene expression in multiple tissue types, including whole blood and liver (Figure 4), indicating that *MST1*,* JAK2*,* MUC19* and *VDR* have synergistic reinforcing action in Crohn's disease development in the Chinese population through interacting with *NOD2*.

### 3.5 Validation of main genetic associations and gene‐gene interactions using ImmunoChip data set

We performed validation analysis of the main genetic effects and gene‐gene interactions identified by target capture sequencing using data from the Hong Kong Chinese ImmunoChip data set comprising 235 controls and 531 patients with IBD, including 388 patients with Crohn's disease, In the ImmunoChip data set, there was no *MUC19* gene marker. Therefore, its main and interaction could not be calculated. For *MST1*, after quality control, there was only 1 SNP in the validation data set, which was restrictive for our analysis. For main effects, *JAK2*,* TNFSF15*,* ZNF365* and *PTPN22* showed consistent small *P*‐values in the original sequencing data as well as the validation with IBD and Crohn's disease data sets (Table S4). For gene‐gene interactions, the top pair *MST1*‐*JAK2* had significant *P*‐values in the original and validation data set (original: *P *=* *9.44 × 10^−11^; validation IBD: *P *=* *7.28 × 10^−5^; validation Crohn's disease: *P *=* *4.81 × 10^−3^). The remaining three pairs that exhibited consistent small *P*‐values were *NOD2*‐*JAK2*,* IL23R*‐*MST1* and *MST1*‐*PTGER4* (Table S5).

## 4 DISCUSSION

Although the prevalence and incidence of Crohn's disease are higher in Western countries, they continue to rise in Asia, especially in China. It is anticipated that the number of cases of IBD in Asia might overtake that of the Western world by 2025.^29^ In this study, we fine‐mapped 23 known Crohn's disease susceptibility genes to identify the causal variants and delineate the relative contribution of these variants to Crohn's disease in the Chinese population. Identification of causal variants is key to understanding the molecular mechanism by which disease susceptibility genes contribute to pathogenesis as well as formulating novel therapeutic strategies. A major advantage of using targeted capture sequencing for fine mapping is the directed focus on genes of interest. Therefore, unlike GWAS, our study was not restricted by the conventional genome‐wide significance threshold as a result of fewer multiple testing.

In the targeted captured regions, the synonymous SNP (ie rs144982232) in *MST1* was most significantly associated with Crohn's disease in the Chinese population. Although synonymous SNPs have long been regarded as inconsequential as the primary sequence of the protein is retained, studies have demonstrated that synonymous SNPs can affect mRNA splicing, stability and structure as well as protein folding.^30^ Through in silico RNA folding prediction (http://rna.urmc.rochester.edu/RNAstructureWeb/Servers/Fold/Fold.html), we found that although the SNP A>G at rs144982232 did not significantly affect the secondary structure of *MST1* mRNA, the corresponding nucleotide change occurred in the loop region of the predicted RNA stem‐loop structure (Figure S3), which is implicated in the control of RNA‐protein complex formation.^31^, ^32^

MST1, also known as MSP, is involved in regulating the innate immune response to infections and cellular stress. It binds to the receptor RON to trigger macrophage chemotaxis and activation.^31^ MST1 is a serum protein that circulates in the blood as an inactive single‐chain precursor (pro‐MST1) comprising two chains, α and β.^34^ *MST1* was first recognized as a Crohn's disease risk gene (odds ratio = 1.20) in a GWAS where a single non‐synonymous SNP rs3197999 corresponding to the amino acid substitution R689C in the β‐chain was identified.^35^ The mechanism of this potential causal variant for Crohn's disease was controversial.^36^, ^37^ One study showed that R689C polymorphism had no impact on the ability of MST1 to bind to or signal through RON, whereas carriers of the 689C polymorphism had lower concentrations of MST1 in their serum, which could possibly increase Crohn's disease risk.^36^ However, another study showed that the affinity to RON of MST1 with the 689C polymorphism was approximately 10‐fold lower than that of the wild‐type MST1 and the thermal stability of the mutant MST1 was slightly lower than that of wild‐type MST1.^37^ However, rs3197999 did not show up as a significant SNP in our study. Instead, the SNP A>G at rs144982232 showed the strongest association with Crohn's disease in our cohort (odds ratio = 4.87; *P *=* *1.78 × 10^−5^). Early GWASs showed that with the exception of *NOD2*, the typical effect size of Crohn's disease susceptibility locus was modest (odds ratio < 1.3).^38^, ^39^ Herein, we reported for the first time a new Crohn's disease susceptibility SNP with a high odds ratio in the Chinese population. Moreover, our data suggested that different causal variants of MST1 might be operative in the Western and the Chinese populations. Nevertheless, it is important to note that our ImmunoChip data set did not cover *MST1* rs144982232 and its association with Crohn's disease has to be consolidated with an independent Chinese cohort.

The genetic heterogeneity between East Asians and Europeans at alleles of large effect could be exemplified by *NOD2*. The contribution of *NOD2* rare variants to risk and site of Crohn's disease was well studied and explained in Caucasians, with 4 mutations (P268S, R702W, G908R and 3020insC) showing the strongest association.^40^, ^41^, ^42^ However, a previous study demonstrated no association of *NOD2* R702W and G908R with IBD in Chinese patients.^43^ In line with previous genetic studies of Crohn's disease in Asians,^1^ a recent study showed that the three coding variants in *NOD2* in Europeans did not exist in East Asians.^21^ Furthermore, no SNP within *NOD2* even attained a suggestive evidence of association in the East Asian cohort, indicating that different genetic factors are operative in the Western and East Asian populations to contribute to Crohn's disease. All these findings prompted us to examine whether *NOD2* could interact with other genes to influence Crohn's disease risk in the Chinese cohort. In this study, by SKAT analysis and *W*‐test, even though *NOD2* SNPs individually were not significantly associated with Crohn's disease, co‐occurrence of *NOD2* rs2111234 and *MST1* rs144982232, *JAK2* rs1159782 or *VDR* rs11574129 attained a significant association. eQTL analysis further substantiated this discovery by showing the strong association between *NOD2* rs2111234 and *NOD2* gene expression, especially in whole blood and spleen. It is tantalizing to postulate that when combined with other IBD SNPs, *NOD2* SNPs could synergistically influence the risk for Crohn's in the Chinese population. It also suggests that the pathogenesis of IBD, in both the West and the East, is likely to be driven by the interplay of an abnormal immune response to gut microbes.

Another example of genetic heterogeneity in different ethnic groups in Crohn's disease pathogenesis is *JAK2*. A recent meta‐analysis demonstrated that *JAK2* rs10758669 was significantly associated with Crohn's disease in Caucasians but not Asians.^44^ Consistently, rs10758669 did not exhibit association with disease status in our cohort. In contrast, another SNP rs1159782 was fine‐mapped to be the most strongly associated SNP in *JAK2* with an odds ratio of 3.72. Given that many JAK inhibitors for IBD are now undergoing phase 3 trials,^45^ it is hopeful that JAK inhibition will benefit this subset of patients with genetic susceptibility in *JAK2*.

From epistasis analysis, we found the most connected gene with other SNPs that synergistically conferred risks to Crohn's disease was *MST1*. In particular, *MST1* rs144982232 showed interactions with other IBD genes, including *JAK2*,* NOD2*,* ATG16L*,* VDR* and *STAT3*, indicating a more complicated role of *MST1* in Crohn's disease pathogenesis. Among these pairs, the top significant interactions were with *JAK2*,* NOD2* and *MUC19*. To this end, *MST1*‐*JAK2* interaction has also been identified in ulcerative colitis,^46^ another major form of IBD. *NOD2* polymorphisms could also modulate innate immune response^47^ whereas *MUC19* deficiency could impair mucus production,^48^ both of which are important for mucosal barrier function and the control of subsequent invasion of commensals or opportunistic pathogens. The co‐involvement of *NOD2*,* JAK2*,* MUC19* and *MST1* in mucosal defence and inflammation in Crohn's disease therefore deserves further study.

Another noteworthy observation is that *VDR* was centred by *MUC19*,* MST1*,* ATG16L1* and *NOD2*, which synergistically contributed to Crohn's disease risk. The association between *VDR* and Crohn's disease has been supported by multiple studies.^49^, ^50^ *VDR*, which codes for vitamin D receptor, is engaged in *NOD2* gene transcription and signalling through *NOD2* to induce expressions of β‐defensin 2 and cathelicidin.^51^ Variants or deletion of *VDR* may also change the microbiota and reduce the host defence through diminishing the production of microbicidal peptides as well as ATG16L1.^52^, ^53^ However, the interaction network among genes of interest that leads to Crohn's disease needs to be elucidated in‐depth in future studies.

Taken together, our data suggested that a novel locus in *MST1* is involved in Crohn's disease in the Chinese population. Interactions between *MST1* and other Crohn's disease susceptibility genes also contribute to disease risk. Future research should focus on resequencing and fine‐mapping analysis to identify causal variants in other Crohn's disease susceptibility genes. Further insights into how different risk alleles interact with each other in different ethnic populations may unravel the complex genetic and environmental influence on IBD and contribute to our understanding of disease pathogenesis.

## CONFLICT OF INTEREST

The authors confirm that there are no conflict of interests.

## AUTHOR CONTRIBUTIONS

WKKW designed the study; WKKW, SHW, MHW and SCN managed the project; RS, YT and MHW conducted the bioinformatic analysis; ZZ and SCN collected clinical specimens; all authors analysed the results; WKKW, RS, ZT and YT wrote the manuscript; SHW, MHW and SCN revised the manuscript and managed the project.
