# Supplementary figures and images for: A novel susceptibility locus in MST1 and gene‐gene interaction network for Crohn's disease in the Chinese population
Source: J Cell Mol Med. 2018 Feb 14;22(4):2368–77. doi: 10.1111/jcmm.13530 (PMC5867068; doi:10.1111/jcmm.13530)

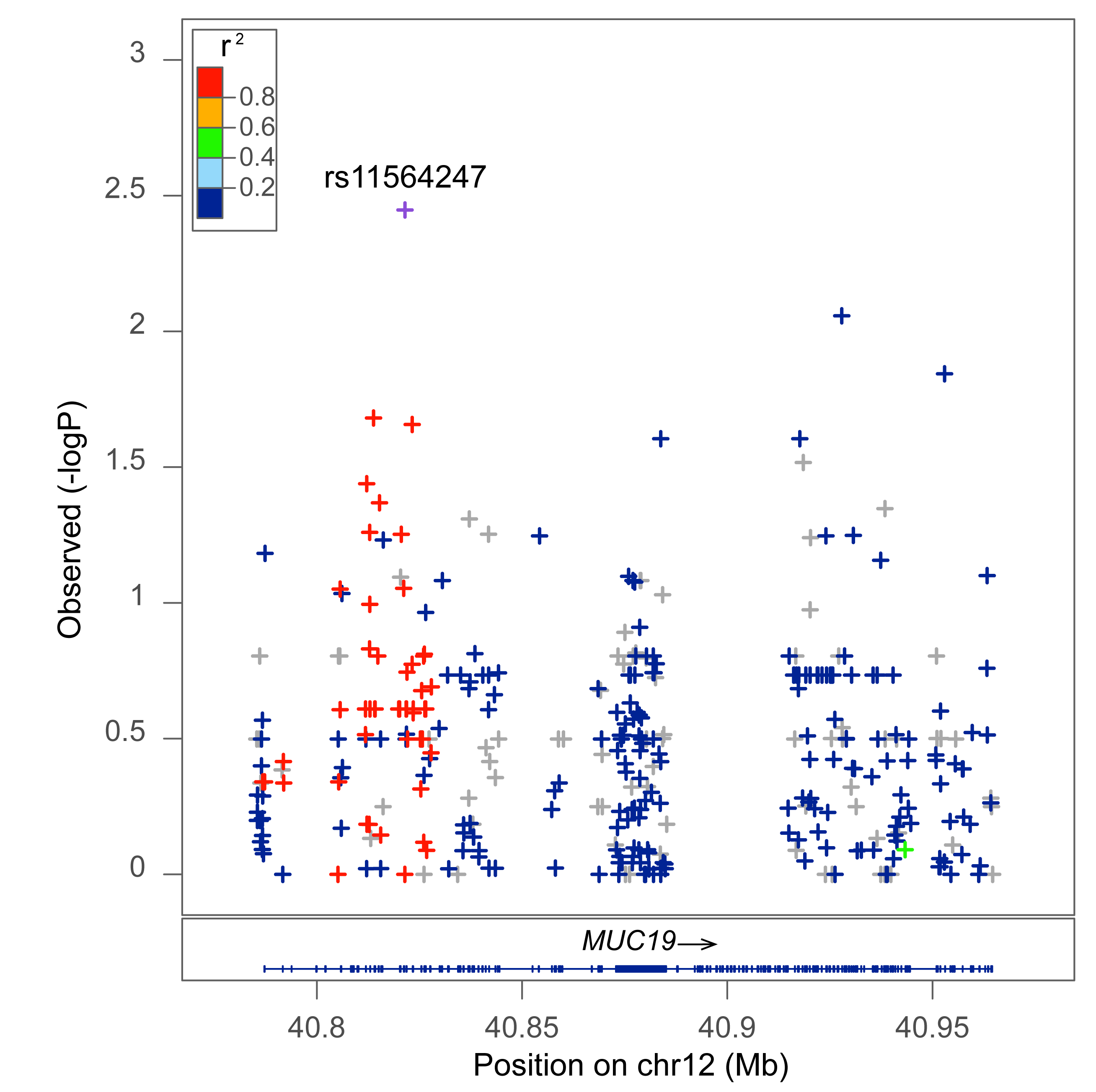

Supplement: Supplementary file 1 [file JCMM-22-2368-s001.tif]

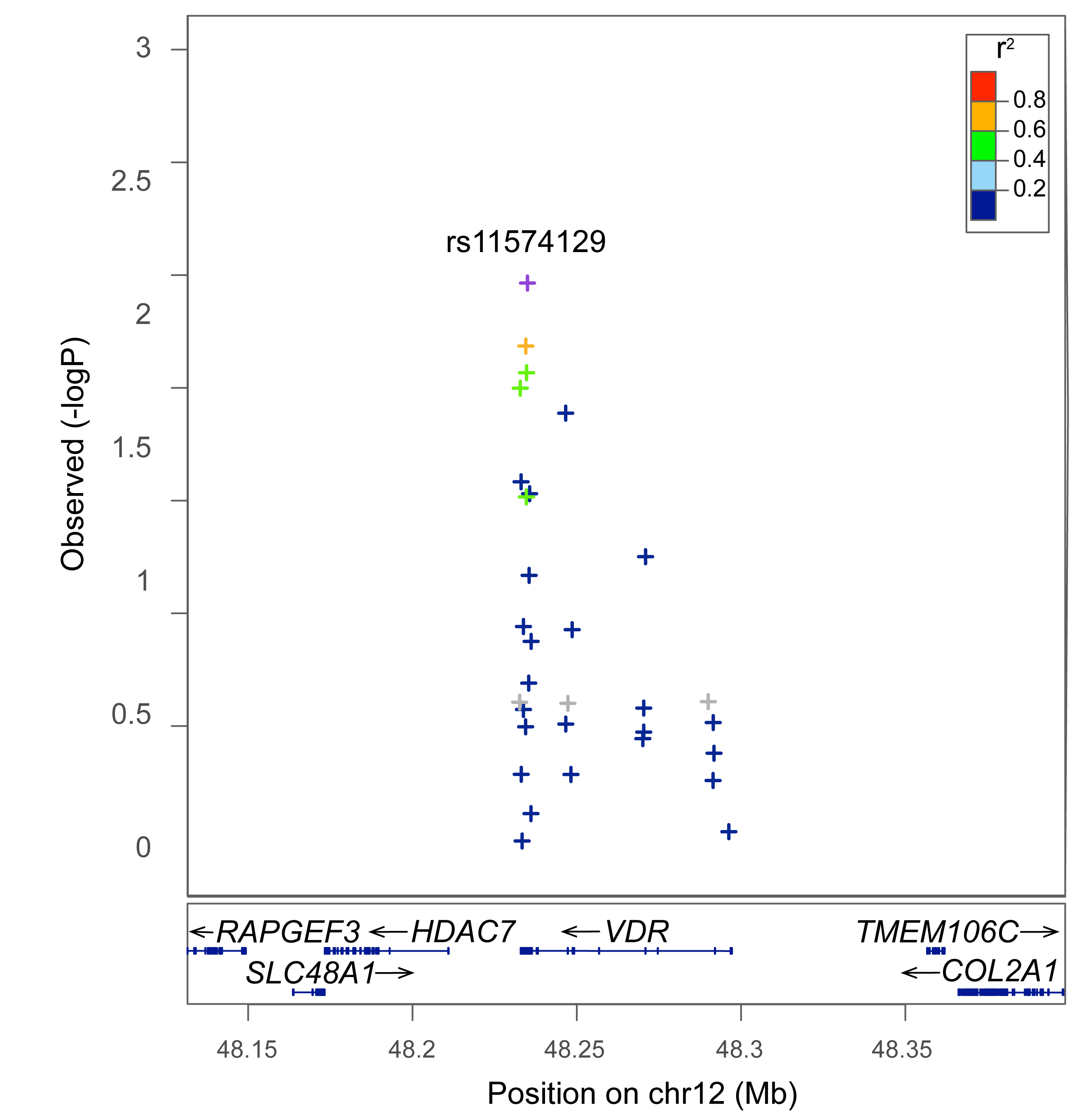

Supplement: Supplementary file 2 [file JCMM-22-2368-s002.tif]

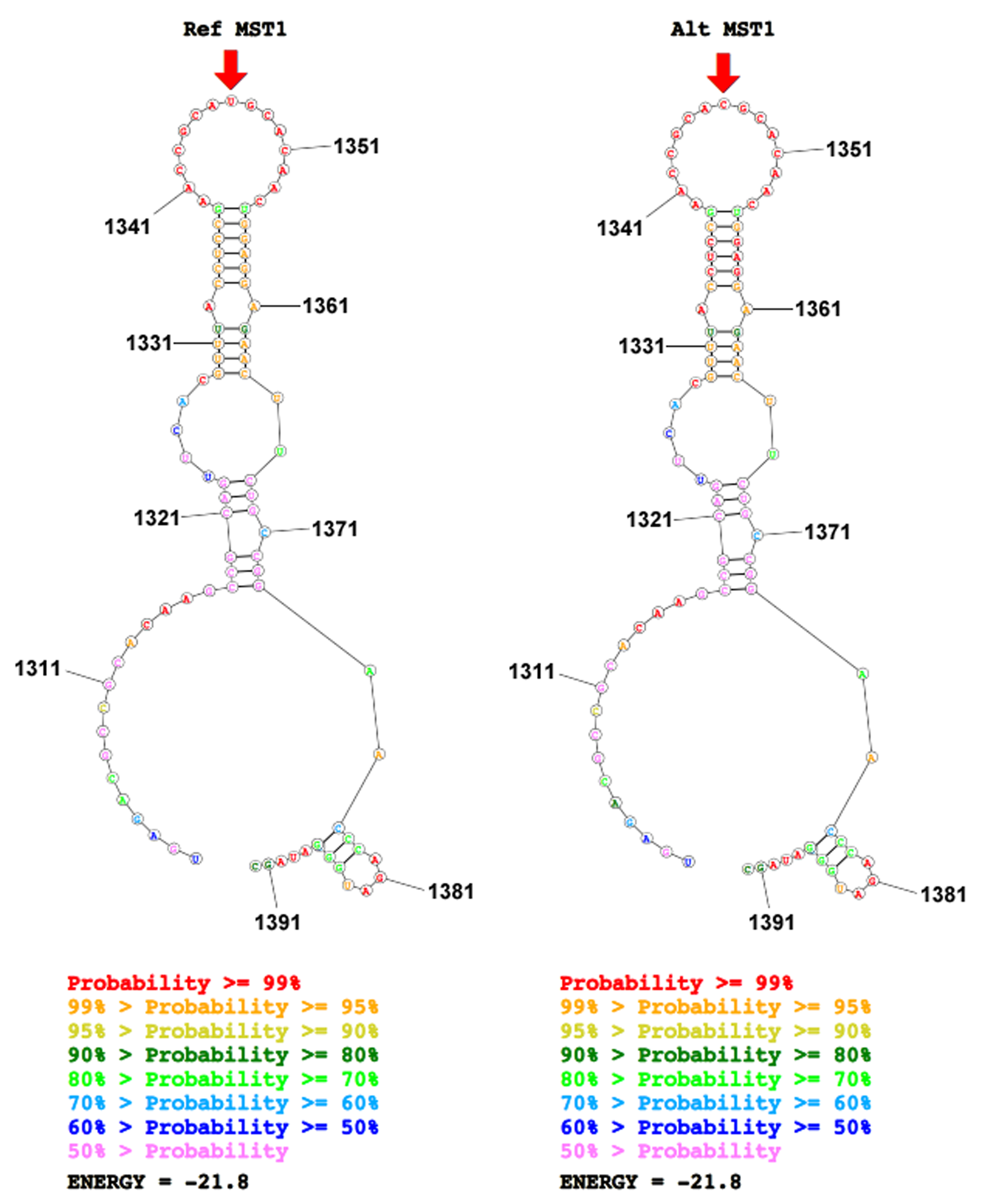

Supplement: Supplementary file 3 [file JCMM-22-2368-s003.tif]
