# Supplementary material for: A novel susceptibility locus in MST1 and gene‐gene interaction network for Crohn's disease in the Chinese population
Source: J Cell Mol Med. 2018 Feb 14;22(4):2368–77. doi: 10.1111/jcmm.13530 (PMC5867068; doi:10.1111/jcmm.13530)
Supplement: Supplementary file 4 [file JCMM-22-2368-s004.doc]

**Supplementary Information**

**Table S1 Demographic data of study participants**

|  | **Case**  **(n = 262)** | **Control**  **(n = 323)** |
| --- | --- | --- |
| **Age** |  |  |
| By male | 43.8 ± 12.5 | 57.1 ± 9.9 |
| By female | 43.3 ± 13.4 | 54.9 ± 10.3 |
| Overall | 43.6±12.9 | 55.9±5.9 |
| **Gender** |  |  |
| Male | 160 (61.1%) | 156 (48.3%) |
| Female | 102 (38.9) | 167 (51.7) |
| **Age of diagnosis** | |  |
| By male | 30.8±13.3 | n/a |
| By female | 31.9±15.2 | n/a |
| Overall | 31.2±13.9 | n/a |

**Table S2 Depth and coverage of targeted capture sequencing**

|  | **Case** | **Control** |
| --- | --- | --- |
| Average sequencing depth on target | 54.62 | 54.15 |
| Average sequencing depth near target | 10.73 | 10.31 |
| Mismatch rate in target region | 0.25% | 0.23% |
| Mismatch rate in all effective sequence | 0.29% | 0.28% |
| Coverage of target region | 99.72% | 99.70% |
| Coverage of flanking region | 95.45% | 94.57% |
| Fraction of target covered with at least 20x | 92.39% | 92.67% |
| Fraction of target covered with at least 10x | 97.68% | 97.70% |
| Fraction of target covered with at least 4x | 99.17% | 99.15% |
| Fraction of flanking region covered with at least 20x | 16.51% | 15.63% |
| Fraction of flanking region covered with at least 10x | 43.67% | 41.98% |
| Fraction of flanking region covered with at least 4x | 74.98% | 72.66% |
| Mapping rate | 99.25% | 99.23% |
| Duplicate rate | 2.52% | 2.14% |

**Table S3 Quality control information**

|  | **# of SNPs** | **# of SNPs removed** | **Cumulatively removed (%)** |
| --- | --- | --- | --- |
| Before QC | 2197 | 0 | 0 |
| Genotype missing percentage > 5% | 2197 | 0 | 0 |
| No variance | 2187 | 10 | 0.46 |
| *HWE *p* < 0.05 | 2046 | 141 | 6.42 |

* *p*-value after Bonferroni correction.

Table S4 Validating main genetic associations with ImmunoChip dataset.

| **Rank** | **SNP** | ***p*-value** | **Gene** | **MAF** | **Odds Ratio** | **Data** |
| --- | --- | --- | --- | --- | --- | --- |
| 1 | rs144982232 | 1.78E-05 | *MST1* | 0.038 | 4.87 | Original |
| 2 | **rs1159782** | **2.34E-04** | ***JAK2*** | **0.053** | **3.72** | **Original** |
|  | **imm_9_5104773** | **4.16E-05** | ***JAK2*** | **0.289** | **1.80** | **V-IBD** |
|  | **imm_9_4988855** | **6.38E-03** | ***JAK2*** | **0.269** | **1.57** | **V-CD** |
| 3 | rs2111234 | 7.59E-04 | *NOD2* | 0.034 | 5.09 | Original |
|  | imm_16_49291360 | 2.99E-01 | *NOD2* | 0.068 | 0.78 | V-IBD |
|  | imm_16_49317481 | 2.30E-01 | *NOD2* | 0.211 | 0.80 | V-CD |
| 4 | **rs7848647** | **1.46E-03** | ***TNFSF15*** | **0.448** | **0.69** | **Original** |
|  | **ccc.9.116595595.C.T** | **2.53E-03** | ***TNFSF15*** | **0.213** | **1.60** | **V-IBD** |
|  | **ccc.9.116595595.C.T** | **4.97E-04** | ***TNFSF15*** | **0.211** | **1.84** | **V-CD** |
| 5 | **rs7915131** | **1.34E-03** | ***ZNF365*** | **0.343** | **0.68** | **Original** |
|  | **rs7094985** | **6.99E-02** | ***ZNF365*** | **0.499** | **1.25** | **V-IBD** |
|  | **imm_10_64066927** | **9.12E-03** | ***ZNF365*** | **0.353** | **0.66** | **V-CD** |
| 6 | **rs1970559** | **3.29E-03** | ***PTPN22*** | **0.041** | **0.39** | **Original** |
|  | **imm_1_114203427** | **2.26E-01** | ***PTPN22*** | **0.161** | **0.81** | **V-IBD** |
|  | **imm_1_114214190** | **3.15E-02** | ***PTPN22*** | **0.046** | **0.48** | **V-CD** |
| 7 | rs2476602 | 4.09E-03 | *PTPN22* | 0.039 | 0.38 | Original |
| 8 | rs4746516 | 2.03E-03 | *ZNF365* | 0.200 | 0.63 | Original |
| 9 | rs7869668 | 3.29E-03 | *JAK2* | 0.417 | 1.44 | Original |
| 10 | rs10822044 | 4.33E-03 | *ZNF365* | 0.231 | 0.68 | Original |

Original: The original target re-sequencing data set

V-IBD: the validation ImmunoChip data using IBD phenotype

V-CD: the validation ImmunoChip data using Crohn’s disease phenotype

**Table S5 Validating gene-gene interactions with ImmunoChip dataset.**

| **Rank** | **SNP1** | **Gene1** | **SNP2** | **Gene2** | **p-value** | **Data** |
| --- | --- | --- | --- | --- | --- | --- |
| 1 | **rs144982232** | ***MST1*** | **rs1159782** | ***JAK2*** | **9.44E-11** | Original |
|  | **imm_3_49696536** | ***MST1*** | **imm_9_5047284** | ***JAK2*** | **7.28E-05** | V-IBD |
|  | **imm_3_49696536** | ***MST1*** | **imm_9_5047284** | ***JAK2*** | **0.00481** | V-CD |
| 2 | rs144982232 | *MST1* | rs2111234 | *NOD2* | 1.79E-09 | Original |
|  | imm_3_49696536 | *MST1* | imm_16_49291470 | *NOD2* | **0.037** | V-IBD |
|  | imm_3_49696536 | *MST1* | imm_16_49317481 | *NOD2* | 0.154 | V-CD |
| 3 | rs144982232 | *MST1* | rs116937891 | *MUC19* | 1.01E-08 | Original |
| 4 | rs144982232 | *MST1* | rs11564247 | *MUC19* | 1.34E-08 | Original |
| 5 | rs144982232 | *MST1* | rs80205770 | *MUC19* | 2.15E-08 | Original |
| 6 | rs144982232 | *MST1* | rs56191322 | *PTPN22* | 3.06E-08 | Original |
|  | imm_3_49696536 | *MST1* | imm_18_12831176 | *PTPN22* | **0.0325** | V-IBD |
|  | imm_3_49696536 | *MST1* | imm_1_114209070 | *PTPN22* | 0.118 | V-CD |
| 7 | rs144982232 | *MST1* | rs191850264 | *MUC19* | 3.11E-08 | Original |
| 8 | rs144982232 | *MST1* | rs2289473 | *ATG16L1* | 4.11E-08 | Original |
|  | imm_3_49696536 | *MST1* | imm_2_233868852 | *ATG16L1* | 2.91E-01 | V-IBD |
|  | imm_3_49696536 | *MST1* | imm_2_233859696 | *ATG16L1* | 1.62E-01 | V-CD |
| 9 | **rs2111234** | ***NOD2*** | **rs1159782** | ***JAK2*** | **5.76E-08** | Original |
|  | **imm_16_49305205** | ***NOD2*** | **imm_9_5012807** | ***JAK2*** | **2.59E-05** | V-IBD |
|  | **imm_16_49291360** | ***NOD2*** | **imm_9_5109939** | ***JAK2*** | **1.55E-03** | V-CD |
| 10 | rs144982232 | *MST1* | rs12370083 | *MUC19* | 6.68E-08 | Original |
| 11 | **rs6687620** | ***IL23R*** | **rs144982232** | ***MST1*** | **6.73E-08** | Original |
|  | **chr1_67421184** | ***IL23R*** | **imm_3_49696536** | ***MST1*** | **2.38E-03** | V-IBD |
|  | **imm_1_67477451** | ***IL23R*** | **imm_3_49696536** | ***MST1*** | **3.07E-02** | V-CD |
| 12 | rs144982232 | *MST1* | rs2229829 | *VDR* | 8.16E-08 | Original |
|  | imm_3_49696536 | *MST1* | rs2239182 | *VDR* | 2.82E-01 | V-IBD |
|  | imm_3_49696536 | *MST1* | rs11168267 | *VDR* | 8.12E-02 | V-CD |
| 13 | rs144982232 | *MST1* | rs11564248 | *MUC19* | 1.14E-07 | Original |
| 14 | rs144982232 | *MST1* | rs2291282 | *STAT3* | 1.22E-07 | Original |
|  | imm_3_49696536 | *MST1* | imm_17_37728406 | *STAT3* | 2.89E-01 | V-IBD |
|  | imm_3_49696536 | *MST1* | imm_17_37745206 | *STAT3* | 3.21E-01 | V-CD |
| 15 | rs144982232 | *MST1* | rs9837520 | *MST1* | 1.23E-07 | Original |
| 16 | rs144982232 | *MST1* | rs78930461 | *ATG16L1* | 1.27E-07 | Original |
|  | imm_3_49696536 | *MST1* | imm_2_233868852 | *ATG16L1* | 2.91E-01 | V-IBD |
|  | imm_3_49696536 | *MST1* | imm_2_233859696 | *ATG16L1* | 1.62E-01 | V-CD |
| 17 | rs144982232 | *MST1* |  | *VDR* | 1.36E-07 | Original |
| 18 | rs144982232 | *MST1* | rs7487333 | *MUC19* | 1.53E-07 | Original |
| 19 | rs144982232 | *MST1* | rs12601611 | *STAT3* | 1.63E-07 | Original |
| 20 | **rs144982232** | ***MST1*** |  | ***PTGER4*** | **1.96E-07** | Original |
|  | **imm_3_49696536** | ***MST1*** | **imm_5_40723816** | ***PTGER4*** | **2.50E-03** | V-IBD |
|  | **imm_3_49696536** | ***MST1*** | **imm_5_40721552** | ***PTGER4*** | **2.99E-02** | V-CD |

Original: The original target re-sequencing data set

V-IBD: the validation ImmunoChip data using IBD phenotype

V-CD: the validation ImmunoChip data using Crohn’s disease phenotype

**Table S6 Allele distribution of top 10 SNPs identified by SKAT among 23 Crohn’s disease susceptibility genes (SNPs presented in Table 1)**

| **Rank** | **SNP** | **Position** | **Gene** | **Control^1^** | | | **Case^1^** | | |
| --- | --- | --- | --- | --- | --- | --- | --- | --- | --- |
|  |  |  |  | AA | Aa | aa | AA | Aa | aa |
| 1 | rs144982232 | 49723141 | *MST1* | 0.973 | 0.027 | 0 | 0.883 | 0.111 | 0.007 |
| 2 | rs1159782 | 5078117 | *JAK2* | 0.963 | 0.027 | 0.010 | 0.883 | 0.070 | 0.047 |
| 3 | rs2111234 | 50734033 | *NOD2* | 0.980 | 0.017 | 0.003 | 0.926 | 0.034 | 0.040 |
| 4 | rs7848647 | 117569046 | *TNFSF15* | 0.238 | 0.537 | 0.225 | 0.359 | 0.477 | 0.164 |
| 5 | rs7915131 | 64418656 | *ZNF365* | 0.362 | 0.503 | 0.134 | 0.473 | 0.453 | 0.074 |
| 6 | rs1970559 | 114377148 | *PTPN22* | 0.893 | 0.097 | 0.010 | 0.953 | 0.047 | 0 |
| 7 | rs2476602 | 114396955 | *PTPN22* | 0.899 | 0.091 | 0.010 | 0.956 | 0.044 | 0 |
| 8 | rs4746516 | 64426056 | *ZNF365* | 0.597 | 0.332 | 0.070 | 0.705 | 0.265 | 0.030 |
| 9 | rs7869668 | 5069837 | *JAK2* | 0.423 | 0.409 | 0.168 | 0.315 | 0.446 | 0.238 |
| 10 | rs10822044 | 64418089 | *ZNF365* | 0.530 | 0.409 | 0.060 | 0.644 | 0.319 | 0.037 |

1: number of controls: 298; Number of cases: 298.

**Table S7 Genotype distribution of top 20 SNP-SNP interactions among 95 significant pairs in 23 genes identified by W-test^1^ (Pairs in manuscript Table 2)**

| Rank | SNP pair information | | Genotype frequency (SNP1, SNP2) | | | | | | | | | |
| --- | --- | --- | --- | --- | --- | --- | --- | --- | --- | --- | --- | --- |
|  | SNP1 | SNP2 | case/control^2^ | (AA,BB) | (AA,Bb) | (AA,bb) | (Aa,BB) | (Aa,Bb) | (Aa,bb) | (aa,BB) | (aa,Bb) | (aa,bb) |
| 1 | rs144982232 | rs1159782 | control | 0.94 | 0.023 | 0.01 | 0.023 | 0.003 | 0 | 0 | 0 | 0 |
|  |  |  | case | 0.782 | 0.06 | 0.04 | 0.094 | 0.01 | 0.007 | 0.007 | 0 | 0 |
| 2 | rs144982232 | rs2111234 | control | 0.953 | 0.017 | 0.003 | 0.027 | 0 | 0 | 0 | 0 | 0 |
|  |  |  | case | 0.812 | 0.03 | 0.04 | 0.107 | 0.003 | 0 | 0.007 | 0 | 0 |
| 3 | rs144982232 | rs116937891 | control | 0.963 | 0.01 | 0 | 0.023 | 0.003 | 0 | 0 | 0 | 0 |
|  |  |  | case | 0.842 | 0.04 | 0 | 0.111 | 0 | 0 | 0.007 | 0 | 0 |
| 4 | rs144982232 | rs11564247 | control | 0.97 | 0.003 | 0 | 0.027 | 0 | 0 | 0 | 0 | 0 |
|  |  |  | case | 0.849 | 0.034 | 0 | 0.107 | 0.003 | 0 | 0.007 | 0 | 0 |
| 5 | rs144982232 | rs80205770 | control | 0.933 | 0.04 | 0 | 0.023 | 0.003 | 0 | 0 | 0 | 0 |
|  |  |  | case | 0.799 | 0.084 | 0 | 0.104 | 0.007 | 0 | 0.007 | 0 | 0 |
| 6 | rs144982232 | rs56191322 | control | 0.966 | 0.007 | 0 | 0.027 | 0 | 0 | 0 | 0 | 0 |
|  |  |  | case | 0.849 | 0.034 | 0 | 0.111 | 0 | 0 | 0.007 | 0 | 0 |
| 7 | rs144982232 | rs191850264 | control | 0.966 | 0.003 | 0.003 | 0.027 | 0 | 0 | 0 | 0 | 0 |
|  |  |  | case | 0.852 | 0.03 | 0 | 0.111 | 0 | 0 | 0.007 | 0 | 0 |
| 8 | rs144982232 | rs2289473 | control | 0.963 | 0.01 | 0 | 0.023 | 0.003 | 0 | 0 | 0 | 0 |
|  |  |  | case | 0.849 | 0.034 | 0 | 0.111 | 0 | 0 | 0.007 | 0 | 0 |
| 9 | rs2111234 | rs1159782 | control | 0.943 | 0.027 | 0.01 | 0.017 | 0 | 0 | 0.003 | 0 | 0 |
|  |  |  | case | 0.812 | 0.07 | 0.044 | 0.03 | 0 | 0.003 | 0.04 | 0 | 0 |
| 10 | rs144982232 | rs12370083 | control | 0.966 | 0.003 | 0.003 | 0.027 | 0 | 0 | 0 | 0 | 0 |
|  |  |  | case | 0.852 | 0.02 | 0.01 | 0.111 | 0 | 0 | 0.007 | 0 | 0 |
| 11 | rs6687620 | rs144982232 | control | 0.966 | 0.027 | 0 | 0.007 | 0 | 0 | 0 | 0 | 0 |
|  |  |  | case | 0.852 | 0.111 | 0.007 | 0.027 | 0 | 0 | 0.003 | 0 | 0 |
| 12 | rs144982232 | rs2229829 | control | 0.963 | 0.01 | 0 | 0.027 | 0 | 0 | 0 | 0 | 0 |
|  |  |  | case | 0.849 | 0.034 | 0 | 0.111 | 0 | 0 | 0.007 | 0 | 0 |
| 13 | rs144982232 | rs11564248 | control | 0.966 | 0.007 | 0 | 0.027 | 0 | 0 | 0 | 0 | 0 |
|  |  |  | case | 0.856 | 0.027 | 0 | 0.111 | 0 | 0 | 0.007 | 0 | 0 |
| 14 | rs144982232 | rs2291282 | control | 0.946 | 0.027 | 0 | 0.027 | 0 | 0 | 0 | 0 | 0 |
|  |  |  | case | 0.822 | 0.057 | 0.003 | 0.107 | 0.003 | 0 | 0.007 | 0 | 0 |
| 15 | rs144982232 | rs9837520 | control | 0.923 | 0.05 | 0 | 0.023 | 0.003 | 0 | 0 | 0 | 0 |
|  |  |  | case | 0.799 | 0.084 | 0 | 0.111 | 0 | 0 | 0.007 | 0 | 0 |
| 16 | rs144982232 | rs78930461 | control | 0.973 | 0 | 0 | 0.023 | 0.003 | 0 | 0 | 0 | 0 |
|  |  |  | case | 0.869 | 0.013 | 0 | 0.111 | 0 | 0 | 0.007 | 0 | 0 |
| 17 | rs144982232 |  | control | 0.973 | 0 | 0 | 0.027 | 0 | 0 | 0 | 0 | 0 |
|  |  |  | case | 0.866 | 0.017 | 0 | 0.111 | 0 | 0 | 0.007 | 0 | 0 |
| 18 | rs144982232 | rs7487333 | control | 0.943 | 0.03 | 0 | 0.027 | 0 | 0 | 0 | 0 | 0 |
|  |  |  | case | 0.822 | 0.06 | 0 | 0.104 | 0.007 | 0 | 0.007 | 0 | 0 |
| 19 | rs144982232 | rs12601611 | control | 0.973 | 0 | 0 | 0.027 | 0 | 0 | 0 | 0 | 0 |
|  |  |  | case | 0.862 | 0.01 | 0.01 | 0.107 | 0 | 0.003 | 0.007 | 0 | 0 |
| 20 | rs144982232 |  | control | 0.97 | 0.003 | 0 | 0.027 | 0 | 0 | 0 | 0 | 0 |
|  |  |  | case | 0.862 | 0.02 | 0 | 0.107 | 0.003 | 0 | 0.007 | 0 | 0 |

1: If a genotype combination has 0 frequency observed in both case and control groups, this cell is not considered and degrees of freedom is reduced accordingly [1]

2: number of controls: 298; Number of cases: 298.

Reference:

[1] Wang, M.H., et al., *A fast and powerful W-test for pairwise epistasis testing.* Nucleic Acids Res, 2016. 44(12): p. e115.
