# Supplementary material for: A novel susceptibility locus in MST1 and gene‐gene interaction network for Crohn's disease in the Chinese population
Source: J Cell Mol Med. 2018 Feb 14;22(4):2368–77. doi: 10.1111/jcmm.13530 (PMC5867068; doi:10.1111/jcmm.13530)
Supplement: Supplementary file 5 [file JCMM-22-2368-s005.doc]

**Supplementary Figure Legends**

**Figure S1** **Regional association plot of *MUC19*.** Among all SNPs within *MUC19*, rs11564247 showed the strongest association with Crohn’s disease.

**Figure S2 Regional association plot of *VDR*.** Among all SNPs within *VDR*, rs11574129 showed the strongest association with Crohn’s disease.

**Figure S3 Predicted secondary structures of mRNA (from nucleotide 1302 to 1392) encoded by wild-type and rs144982232 A>G *MST1*.** The position of the polymorphic site is indicated with an arrow.
